# The symbolic efficacy of medicinal plants: practices, knowledge, and religious beliefs amongst the Nalu healers of Guinea-Bissau

**DOI:** 10.1186/s13002-016-0095-x

**Published:** 2016-06-17

**Authors:** Amélia Frazão-Moreira

**Affiliations:** Centre for Research in Anthropology, Faculty of Social Sciences and Humanities, Universidade Nova de Lisboa, Lisbon, Portugal

**Keywords:** Healers, Traditional medicine, Islam, Medicinal plants, Ethnopharmacological knowledge, Cosmologies, Guinea-Bissau, Africa

## Abstract

**Background:**

In attempting to understand how the use of medicinal plants is symbolically valued and transformed according to specific cosmologies, we gain valuable insight into the ethnopharmacologial practices, in terms of the major role played by healers, as custodians of local ethnobotanical knowledge, but also as ritual masters.

Thus, the goal of this paper is to understand how medicinal plants are used differently depending on a combination between the healers’ field of expertise and personal history on the one hand, and the diversified religious and symbolical frameworks on the other.

**Methods:**

This essay is based on intense ethnographical research carried out amongst the Nalu people of Guinea-Bissau. Methods included participant observation and semi-directed interviews with six locally-renown healers (four men and two women). The progress of their work and the changes operated within the sets of beliefs associated with ethnopharmacological practices were registered by means of repeated field visits.

**Results:**

A total of 98 species and 147 uses are accounted for, as well as a description of the plant parts that were used, as well as the methods of preparation and application according to the different healers’ specialized practices. At the same time, this research describes those processes based on pre-Islamic and Muslim cosmologies through which medicinal plants are accorded their value, and treatments are granted their symbolic efficiency.

**Conclusions:**

Medicinal plants are valued differently in the pre-Islamic medicine and in the medicine practiced by Islamic masters. The increasing relevance of Islam within this context has affected the symbolic framework of ethnopharmacological practices. Nevertheless, the endurance of those processes by which symbolic efficiency is attributed to local treatments based on plants is explained not only by the syncretic nature of African Islam, but also by the fact that patients adopt different therapeutic pathways simultaneously.

## Background

In the West African rural contexts we can observe the daily collection and distribution of vegetable products for medicinal use. In poor countries, where public health systems are under-resourced, the practices of traditional medicine are of great importance. This is certainly the case of Guinea-Bissau [1, 2].

The Guinea-Bissau traditional medicinal systems are largely based on the use of plants for pharmacological use. Some studies on medicinal plants have been carried out in different ethnic groups, namely Fulani [3] and Bijagó [4, 5]. However, those studies were mainly focused on botanical [6–9] or pharmacological analysis [3, 10, 11]^1^.

This paper aims to present the uses of medicinal plants in the context of Nalu people (southern Guinea-Bissau) based on an ethnobotanical framework that focuses on the connection between plant uses and symbolic aspects. The proposed goal is to understand rationalities, cognitive processes and behaviors involving ethnopharmacological practices, while striving not to take local knowledge out of context, but instead to reflect on how it was generated, transformed, and connected to specialized practices and cosmological beliefs [12]. As Ingold suggests, local traditional knowledge ‘is continually generated and regenerated within the contexts of people’s skilled, practical involvement with significant components of the environment’; knowledge ‘subsists in practical activities themselves, activities that may also be interpreted as ways of remembering’ ([13], p. 307–8). In indigenous conceptualization, knowledge is not understood as ‘a kind of substance’ but rather as ‘a kind of process’.

On the other hand, this paper places its main focus on the healers, as detainers of ethnopharmacological knowledge and guarantors of the population’s survival. Thus, the main focus will be the subjects and their experience of the treatment and cure processes, rather than the medicinal plants *per se*.

Local treatment and cure processes include: cultural representations of health and illness; local healers’ knowledge; nature and world-view perceptions shared by healers and patients; rituals and symbols that legitimize the efficacy of treatments. These elements must be taken together to understand the emic processual efficacy [14]. Said processes imply the coexistence of ancestral local practices and global dynamics of change. We can find different, complementary and simultaneous therapeutic practices in any African culture (e.g. [15–18]).

Based on these assumptions, the present work addresses two main questions: a) to what extent does the Nalu healers’ use of the same plants vary according to their expertise and personal history; b) what role do cosmological perceptions and religious beliefs play in the use of medicinal plants.

Our goal is not providing a description of the ritual or magical uses of plants, but rather of the magical processes that confer symbolical efficacy on the medicinal plants which are not used in ritualized cures. Even though healers and patients alike share a common worldview, the precise and detailed features of said processes, or in other words, the specific actions performed by healers to endow the plants with symbolical efficacy, are in most cases not known to the patients.

This means that while a study focused on the plants and the observation of their therapeutic uses reveals two distinct types of plants used in the African medicinal systems – non ritual plants, used in non-religious treatments, and ritual plants used in ritualistic treatments (e.g. [19, 20]), when we turn to the processes and practices through which healers confer efficacy on the plants, that distinction disappears. Thus, the working hypothesis for this paper is that, amongst the Nalu the distinction between the practical sphere comprised by the knowledge of certain plants’ beneficial properties, and the magical sphere of supernatural intervention needed to render those properties effective, is meaningless from an emic point of view.

On the other hand, we argue that the means used to confer symbolical efficacy upon the treatments using medicinal plants, are different for Nalu healers who summon non-Muslim supernatural entities and those who invoke the words of the Quran.

### Population study

**Fig. 1:**
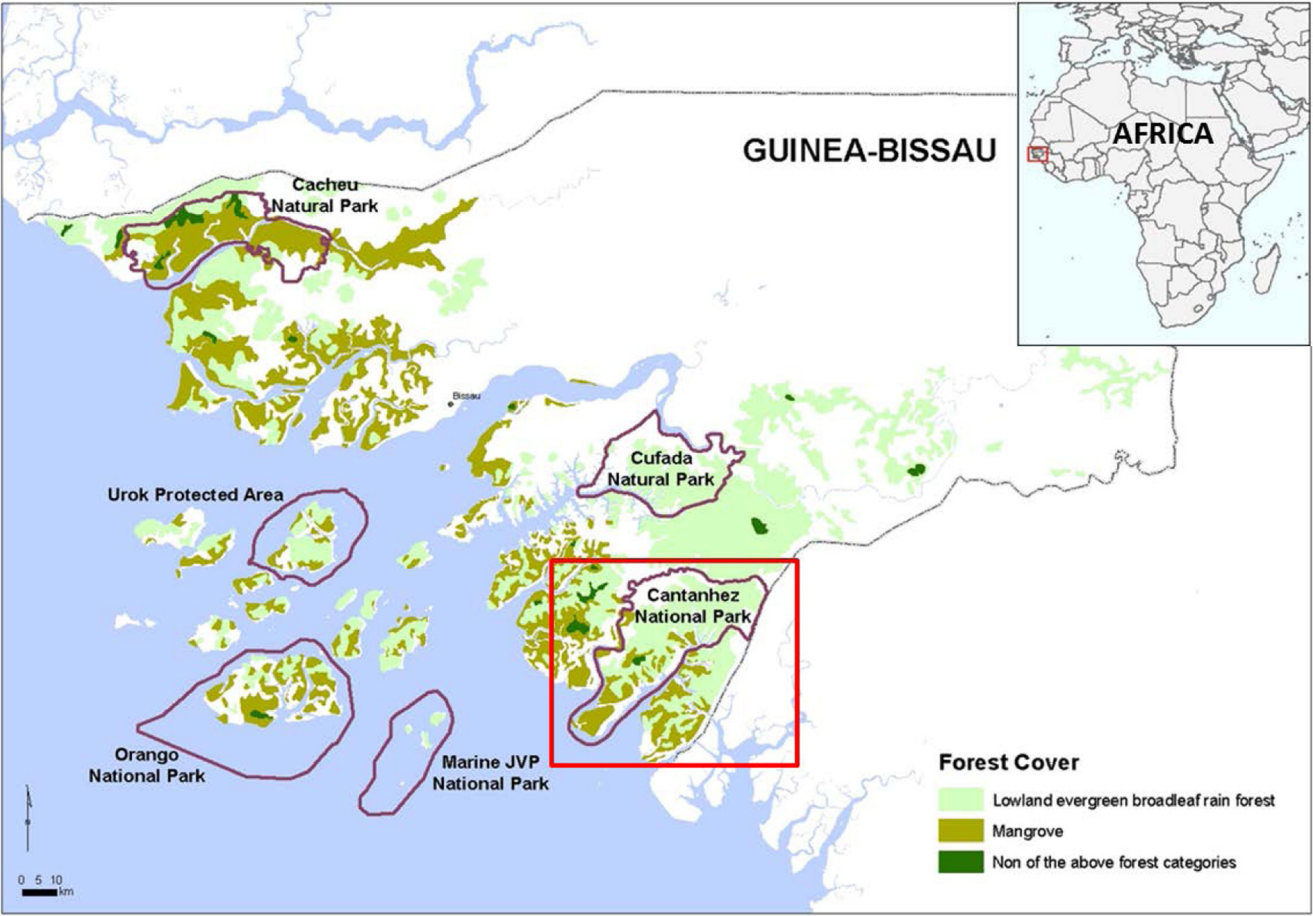
Localization of Guinea-Bissau and the Catanhez National Park

The Nalu people inhabit Guinea-Bissau and Republic of Guinea. The community under analysis is located in Guinea-Bissau in the Cantanhez National Park (Fig. 1).

Nalu economy relies essentially on agriculture, more specifically on rice-growing in flooded areas and shifting cultivation, according to the traditional African slash-and-burn system, as well as on the trade in fruit products and palm oil. Various ecosystems are identified locally as: forest (*n’koi*); new slash-and-burn field (*kakoi*); savannah (*m’báké*); mangrove (*n’kim*); rice-swamp (*n’dále*) and palm tree grove (*n’búm*) [21].

Similarly to other African social organizations, the ordering of productive and reproductive resources, and consequently political power, is mainly influenced by lineage. Together with kinship, distinctions of gender and principles of seniority are crucial to their social and economic organization. In terms of Religion, the Nalu of Cantanhez were Islamized at the beginning of the 20th century [22, 23], and while pre-Islamic beliefs and practices persisted among the Nalu until the turn of the 20th century, surviving in co-existence with Islamic practices until recently, currently they are in the process of being substituted altogether by Islamic-based practices.

## Methods

The data presented was gathered in the course of a broader research, whose aim was to understand the process of the social appropriation of nature and plant classification [24]. An ethnographic methodology was used in this study. The procedures for gathering information on the medical practices and knowledge of the Nalu were participant observation and semi-directed interviews [25, 26]. The present work is based on the information shared by six healers: two old women, two old men, and two younger men. For the most part, the information was obtained during plant-collecting in the forest and savannah, or during the preparation of medicines in the villages. All healers agreed, through prior verbal informed consent, to participate in the study and for their image to appear in scientific publications. The preliminary data collected were reported to a local NGO [27], and final data and later publications were returned to the community.

Initial fieldwork amounted to a total of 16 months, and was conducted throughout the period between 1993 and 1996. More recent field missions, in 2008, 2009, 2012 and 2013, made it possible to revisit the ethnographic setting and confirm the progress of the healers’ work. Arafan Sané and Tcherno Camará, two of the well-reputed men healers in the region, have since passed away. In both cases, one of their male children – Iafai Sané and Amará Camará respectively – occupied their place, thus ensuring that at least part of their knowledge of medicinal plants will endure.

Herbarium vouchers were collected with informants, preserved according to the usual herbarium techniques, and the specimens deposited in the herbarium of IICT (Tropical Research Institute), Lisbon (LISC). The collection of data was made according to the national and international guidelines, with the support and agreement of local communities and Guinea-Bissau institutions (Iniciativa de Cantanhez and, after 2008, Instituto da Biodiversidade e das Áreas Protegidas). Identification of the specimens was based on a complete taxonomic study using Flora of West Tropical Africa [28] and through comparison with specimens already identified in the LISC Herbarium, which possesses the most important collection of specimens from the Guinea-Bissau. Plant names have been checked and updated through online data provided by the Royal Botanic Gardens, Kew and Missouri Botanical Garden (www.theplantlist.org).

The ethnopharmacological and ethnographic information was subjected to a qualitative analysis (e.g. [26, 29]).

## Results and discussion

### Local agents and perceptions of illnesses

Our observations enable us to assert that in the Nalu communities, different agents coexist in the treatment of different illnesses: healers who are specialized in the treatment of specific ailments, mostly physical disturbances, without resource to divination; *djambakus* (healers who are diviners and make ritualized treatments); *marabouts* (Muslim medicine-men); and even ordinary members of the social group, especially the elderly.

Despite the fact that any “classification of sicknesses” is constructed and reconstructed according to context, and that the links between nosological symptoms and categories - as between kinds of sickness and therapeutics -, are not reciprocal [15, 16, 30], after systematizing local medical conceptions we can say that sicknesses fall essentially into two categories: “simple” sicknesses - manifestations of various physical symptoms-, and “*irán* sicknesses”. *Iráns* are supernatural beings that populate the Nalu imagery, and *irán* diseases are disturbances, most of them of psychic nature, believed to be provoked by these spirits.

In the treatment and cure of the illnesses, different symbolic and cosmological frameworks are called forth, both pre-Islamic and Muslim beliefs. Healers summon the power of the spirits (*iráns*), which is essential to their cosmological explanation of the world and nature, whereas for Muslim medicine-men (*marabouts*) the effectiveness of the remedies resides basically in its association with the “sacred word” of the Quran.

Another important aspect derives from the fact that the infirm pursue different therapies and visit several agents of traditional medicine while also - and often simultaneously – seeking the help of state health services, based on the belief in the effectiveness of modern medicine (“the white man’s medicine”), following the very same process that was theorized by authors who examined African medicine, such as Augé [15] and Fassin [16].

### Specialized knowledge of medicinal plants

One hundred and forty seven different uses derived from ninety eight species were shared by healers. They are listed and ordered by healer and by the sicknesses treated by the plants used (Table 1).

**Table 1:** Plant used by healers for medicinal purposes

Family	Genus and species	Nalu name	Voucher^a^	Healer^b^	Ailment	Part of plant used	Preparation	Application
Acanthaceae	*Asystasia gangetica* (L.) T.Anderson		Moreira 85	UC	open wounds	bark	scrape	poultice
	*Anacardium occidentale* L.	ialikê	Moreira 213	TC/DC	lower back pain	leaves	none	topical
	*Mangifera indica* L.	n’mango	Moreira 32	TC/DC	scabies	leaves	macerate	topical
	*Sorindeia juglandifolia* (A.Rich.) Planch. ex Oliv.	n’txaluas	Moreira 154	FB	children’s disease	leaves	macerate	drink
	*Spondias mombin* L.	n’fal	Moreira 24	TC/DC	eye pains	leaves	macerate	wash
Anisophylleaceae	*Anisophyllea laurina* R.Br. ex Sabine	n’sunt	Moreira 57	TC/DC	eye pain	leaves	moisten	topical
Annonaceae	*Annona glabra* L.	n’bonhé	Moreira 38	FB	stomach ache	root	infusion or macerate	drink
	*Annona muricata* L.	n’sóp sóp	Moreira 73	DC	stomach ache	leaves	boil	drink
	*Uvaria chamae* P.Beauv.	n’pinden	Moreira 155	UC	haemorrhoids	root	boil	drink
				AS	open wound	leaves	triturate	topical
	*Xylopia aethiopica* (Dunal) A. Rich.	n’sél	Moreira 168	FB	stomach ache	fruit	boil	drink
Apocynaceae	*Alstonia congensis* Engl.	ianke	Moreira 27	TC/DC	fatigue	bark	macerate	drink
					swelling	latex	none	topical
	Cf. *Strophanthus hispidus* DC.	n’fam	Moreira 78	UC	haemorrhoids	root	peel	swallow
					intestinal parasites	root	peel	swallow
	*Holarrhena floribunda* (G.Don) T.Durand & Schinz	metxel	Moreira 86	UC	stomach ache venereal disease	bark latex	boil scrape	drink swallow
	*Landolphia dulcis* (Sabine ex G.Don) Pichon	urém	Moreira 176	FB	pregnancy	leaves	macerate	drink
	*Landolphia heudelotii* A.DC.	m’bolé	Moreira 158	AS	diarrhoea	leaves	boil	drink
					swelling	leaves	boil	drink
				FB	swollen limbs	leaves	boil	drink
				UC	itching	leaves	boil	wash
	*Rauvolfia vomitoria* Afzel.	n’ti kambirás	Moreira 14	TC/DC	impotence	stem	boil	drink
					stomach ache	root	boil	drink
	*Saba senegalensis* (A.DC.) Pichon	n’badak	Moreira 64	TC/DC	fever	leaves	infusion	wash
	*Tabernaemontana africana* Hook.	n’lat laté	Moreira 15	TC/DC	venereal disease	stem	macerate	drink
Araceae	*Anchomanes difformis* (Blume) Engl.	n’denkamdudu	Moreira 286	AS	infertility	rhizome	boil	drink
	*Cercestis afzelii* Schott	mandonha	Moreira 92	UC	impotence	leaves	boil	drink
Cannabaceae	*Trema orientalis* (L.) Blume	n’robta kabafar	Moreira 114	AS	cough	bark	none	swallow
Capparaceae	*Capparis erythrocarpos* Isert	néeu	Moreira 89, 120	AS	headache	root	triturate	topical
				UC	headache	root	scrape	topical
	*Maerua duchesnei* (De Wild.) F.White	maéf	Moreira 90, 314	AS UC	lower back pain sprains	leaves leaves	triturate triturate	poultice poultice
Caricaceae	*Carica papaya* L.	n’pápa	Moreira 22	TC/DC	risk of miscarriage	root	boil	drink
Celastraceae	*Salacia senegalensis* (Lam.) DC.	mankidés	Moreira 6	TC/DC	tooth decay	root	triturate	topical
Chrysobalanaceae	*Parinari excelsa* Sabine	n’lut	Moreira 145, 196	FB	stomach ache	bark	boil	drink
				TC/DC	cough	bark	macerate	swallow
					open wound	bark	triturate	topical
Combretaceae	*Combretum micranthum* G.Don	n’babass	Moreira 81, 200	TC/DC	scabies	root	triturate	topical
					diabetes	leaves	boil	drink
				UC	scabies	root	scrape	topical
					fever	leaves	boil	drink
	*Guiera senegalensis* J.F.Gmel.	manáf náfen	Moreira 173	AS	open wound	leaves	triturate	poultice
	*Terminalia macroptera* Guill. & Perr.	n’kone	Moreira 126	AS	chest pain	leaves	boil	drink and wash
Compositae	*Aedesia glabra* (Klatt) O.Hoffm.	n’tonpin-na	Moreira 262	AS	stomach and body aches	root	boil	drink
	*Gymnanthemum coloratum* (Willd.) H.Rob. & B.Kahn	n’konkone	Moreira 95	UC	scabies	leaves	macerate	wash
Connaraceae	*Agelaea pentagyna* (Lam.) Baill.		Moreira 190	HBC	scabies	leaves	none	ointment
	*Cnestis ferruginea* Vahl ex DC.	n’xéte nembel	Moreira 16	TC/DC	headache	leaves	macerate	wash
Costaceae	*Costus afer* Ker Gawl.	mabôbé	Moreira 93, 122	UC	eye pain	stem	warm and squeeze	topical
				AS	eye pain	stem	warm and squeeze	topical
Crassulaceae	*Kalanchoe crenata* (Andrews) Haw.	n’txatxatxé	Moreira 242	HBC	newborn baby’s navel	leaves	moisten and squeeze	topical
Cyperaceae	*Cyperus articulatus* L.	n’téd	Moreira 121	AS	newborn with a cough and stomach ache	root	triturate and macerate	drink
	*Scleria racemosa* Poir.	n’txapen ka basop	Moreira 94	UC	pregnancy	leaves	boil	drink
Ebenaceae	*Diospyros heudelotii* Hiern	n’txambortá	Moreira 76	DC	swollen limbs	leaves	macerate	drink and wash
Euphorbiaceae	*Alchornea cordifolia* (Schumach. & Thonn.) Müll.Arg.	n’sum-na	Moreira 4, 129	AS	chest ache	stem	peel	wash
					intestinal parasites	root	boil	drink
					risk of miscarriage	root	boil	drink
				TC/DC	stomach ache	leaves	macerate	drink
	*Manihot esculenta* Crantz	n’mandiok	Moreira 23	TC/DC	eye complaints	leaves	macerate	wash
Hypericaceae	*Psorospermum glaberrimum* Hochr.		Moreira 124	AS	body aches	leaves	boil	drink
Icacinaceae	*Icacina oliviformis* (Poiret) J.Raynal	n’putmé	Moreira 162	FB	children’s disease	leaves	boil	drink
Lamiaceae	*Clerodendrum splendens* G.Don	manar balé	Moreira 48	TC/DC	snake bite	root	macerate	swallow
					contraception	bark and stem	none	drink
	*Premna hispida* Benth.	n’lum bafai	Moreira 68,	HBC	pregnancy	leaves	boil	drink
			110, 170	DC	haemorrhoids	leaves	macerate	drink
				FB	haemorrhoids	leaves	boil or macerate	drink
	*Vitex madiensis* Oliv.	n’sokór	Moreira 177	FB	pregnancy	leaves	macerate	drink
Leguminosae	*Albizia zygia* (DC.) J.F.Macbr.	masanp n’buk	Moreira 172	FB	palpitations	leaves	triturate	swallow
	*Bauhinia thonningii* Schum.	n’bukui	Moreira 30, 105	TC/DC	diarrhoea	leaves	triturate and macerate	drink
					haemorrhoids	bark	boil	drink
				UC	haemorrhoids	bark	boil	drink
	*Caesalpinia benthamiana* (Baill.) Herend. & Zarucchi	n’pinkid sé	Moreira 51, 119	AS	eye complaints	leaves	macerate	topical
				TC/DC	open wound	root and leaves	triturate	topical
	*Cassia sieberiana* DC.	n’sansan	Moreira 33	AS	body aches	root	macerate	drink
				TC/DC	body aches	root	macerate	drink
					impotence	root	macerate	drink
	Cf. *Abrus precatorius* L.		Moreira 83	UC	impotence	leaves	none	swallow
	*Desmodium velutinum* (Willd.) DC.	rap rap	Moreira 21	TC/DC	cholera	leaves and root	macerate	drink
	*Detarium microcarpum* Guill. & Perr.	m’béta	Moreira 63	TC/DC	uncertain	bark	macerate	wash
	*Dialium guineense* Willd.	n’bim	Moreira 52	TC/DC	measles	leaves	boil	drink
	*Dichrostachys cinerea* (L.) Wight & Arn.	n’pinkid úne	Moreira 46, 96	TC/DC	headache rheumatism	bark bark	peel peel	topical topical
				UC	bone pains	bark	peel	topical
	*Erythrina senegalensis* DC.	n’txákarfatch	Moreira 47,	FB	sore throat	bark	boil	drink
			175		palpitations	root	peel and macerate	drink
				TC/DC	pregnancy	bark	peel and boil	drink
	*Parkia biglobosa* (Jacq.) G.Don	n’iú	Moreira 164	FB	treponematoses rheumatism	leaves leaves	triturate triturate	poultice poultice
				TC/DC	stomach ache	bark	boil	drink
	*Pterocarpus erinaceus* Poir.	n’sia	Moreira 9	TC/DC	cough, chest ache	bark	boil	drink
	*Samanea dinklagei* (Harms) Keay	masamp	Moreira 152	DC	lower back pain	root	boil	drink and wash
Loganiaceae	*Usteria guineensis* Willd.	n’átá uóké	Moreira 293	AS	lower back pain	leaves	triturate	poultice
Malvaceae	*Ceiba pentandra* (L.) Gaertn.	n’kauuê	Moreira 59	TC/DC	open wound	bark	triturate	topical
	*Cola nitida* (Vent.) Schott. & Endl.	n’kola	Moreira 72	DC	venereal disease	leaves	burn and boil	topical and wash
	*Hibiscus sterculiifolius* (Guill. & Perr.) Steud.	n’fakaf	Moreira 75	DC	influenza lice	leaves leaves	macerate macerate	drink wash
	*Sida acuta* Burm.f.	n’téksen	Moreira 10	TC/DC	lice	leaves	macerate	wash
Menispermaceae	*Cissampelos mucronata* A.Rich.	n’néo fáfak	Moreira 40	TC/DC	stomach ache	root	macerate	drink
	*Triclisia patens* Oliv.	manar kambantxô	Moreira 132	HBC	expectant mother’s lack of milk	leaves	macerate	drink
Moraceae	*Ficus exasperata* Vahl	n’txéf	Moreira 197	TC/DC	impotence	leaves	macerate	drink
	*Ficus* aff. *umbellata* Vahl	n’fór	Moreira 84	TC/DC	toothache	stem	scrape	topical
				UC	lower back pain	leaves	triturate	topical
	*Ficus sur* Forssk.	n’tonkindjá	Moreira 128, 131	AS	lower back ache	root	triturate and boil	poultice
				FB	failure to menstruate	root	macerate	drink
				TC/DC	stomach ache	bark	macerate	drink
Myrtaceae	*Psidium guajava* L.	goiaba	Moreira 66	TC/DC	dysentery	leaves	boil	drink
Passifloraceae	*Smeathmannia pubescens* Sol. ex R.Br.	n’baptame	Moreira 337	AS	stomach ache venereal disease	root root	boil boil	drink drink
Phyllanthaceae	*Bridelia micrantha* (Hochst.) Baill.	n’tak	Moreira 171, 261	AS	stomach ache	root	boil	drink
				FB	haemorrhoids	leaves	triturate and macerate	drink
	*Hymenocardia acida* Tul.	matik sé	Moreira 166	FB	treponematoses	leaves	paste	poultice
					rheumatism	leaves	paste	poultice
				TC/DC	temporary blindness	leaves	macerate	wash
	*Phyllanthus muellerianus* (Kuntze) Exell	mafér	Moreira 148	FB	pregnancy	leaves	boil	drink
Plantaginaceae	*Scoparia dulcis* L.	n’txinké	Moreira 109	UC	eye pain	leaves	macerate	topical
					children’s stomach ache	leaves	boil	drink
Poaceae	*Oxytenanthera abyssinica* (A.Rich.) Munro	n’fon	Moreira 87	UC	abortion post-natal problems	leaves leaves	boil boil	drink drink
Rubiaceae	*Aidia genipiflora* (DC.) Dandy	n’armass	Moreira 335	AS	impurity	root	foam	wash
					pregnancy	leaves	macerate	drink
	*Canthium* sp.		Moreira 165	FB	treponematoses	leaves	paste	poultice
					rheumatism	leaves	paste	poultice
	*Crossopteryx febrifuga* (Afzel. ex G.Don) Benth.		Moreira 174	FB	pregnancy	leaves	macerate	drink
	*Gardenia ternifolia* subsp. *jovis-tonantis* (Welw.) Verdc. var. *jovis tonantis*	n’dué	Moreira 125	AS	impurities	root	foam	wash
	*Morinda chrysorhiza* (Thonn.) DC.	n’tunké	Moreira 118	AS TC/DC	post-natal post-natal	leaves leaves	boil boil	drink wash
	*Morinda morindoides* (Baker) Milne-Redh.	n’txéf kam nhalankôk	Moreira 113	AS	body aches	leaves	boil	fumigate
	*Pavetta corymbosa* (DC.) F.N.Williams		Moreira 269	HBC	open wound	bark	peel and triturate	topical
	*Psychotria peduncularis* (Salisb.) Steyerm.	n’tokoi	Moreira 161	AS	open wound	leaves	heat	topical
				FB	children’s disease	leaves	boil	drink
	*Sarcocephalus latifolius* (Sm.) E.A.Bruce	n’fol	Moreira 82	AS	intestinal parasites	root	scrape and macerate	drink
				HBC	constipation	leaves	boil	drink
				TC/DC	stomach ache	root	peel and macerate	drink
				UC	pregnancy	leaves	macerate	drink
Rutaceae	*Citrus limon* (L.) Osbeck	n’sinim nelbéne	Moreira 67	TC/DC	scabies	leaves	boil	wash
					venereal disease	leaves and root	boil	drink
					chronic open wound	fruit	moisten	topical
					haemorrhoids	fruit	boil	drink
	*Zanthoxylum leprieurii* Guill. & Perr.	mabár	Moreira 104	UC	risk of miscarriage	root	peel and macerate	drink
Sapindaceae	*Allophylus africanus* P.Beauv.		Moreira 160	HBC	pregnancy	leaves	boil	drink
				FB	swollen limbs	leaves	macerate	drink and wash
	*Lecaniodiscus cupanioides* (Planch.) ex Benth.	n’sonran	Moreira 80	UC	cough	root	peel, triturate and macerate	drink
					hoarseness	root	peel, triturate and macerate	drink
	*Paullinia pinnata* L.	n’fankok	Moreira 76	HBC	diarrhoea in children	leaves	macerate	drink
				FB	weakness in children	leaves	macerate	drink and wash
				UC	scabies	leaves	boil	wash
Smilacaceae	*Smilax anceps* Willd.	n’pôrkam nunpun	Moreira 298	AS	body aches	root	boil	drink
Thymelaeaceae	*Dicranolepis disticha* Planch.	n’saldendek	Moreira 97	UC	intestinal parasites	root	peel, triturate and macerate	drink
Vitaceae	*Cissus rufescens* Guill. & Perr.		Moreira 232	FB	swollen limbs	leaves and root	peel and triturate	topical
Zingiberaceae	*Aframomum alboviolaceum* (Ridl.) K.Schum.	mabôbé	Moreira 115	AS	stomach ache	root	boil	drink
Unidentified		mabobese tchill	Moreira 74	DC	children’s disease	root and leaves	boil and paste	drink and poultice
		méné	Moreira 41	TC/DC	swollen limbs	leaves	boil	wash and fumigate
		n’pitió	Moreira 28	TC/DC	dental caries	bark	triturate	topical
		n’timé	Moreira 153	FB	children’s disease	leaves	macerate	drink
		olo	Moreira 35	TC/DC	pulmonary complaints	leaves	macerate	drink

^a^ Voucher: plant specimens are deposited in LISC Herbarium (IICT -Tropical Research Institute, Lisbon)

^b^ Healer: *AS* Arafan Sane, *DC* Dgibi Camará, *FB* Fatu Bangorá, *HBC* Hajia Bintu Camará, *TC* Tcherno Camará, *TC/DC* Tcherno Camará and Dgibi Camará, *UC* Usumane Cassamá

There are certain medicinal plants known to all the Nalu including the children, such as *n’fol* (*Sarcocephalus latifolius*). But only a few amongst the elder members can identify the ailments that these plants can cure, and only healers know which parts of the plants must be used, as well as the specific combinations, preparation and dosing that apply in each particular circumstance.

The knowledge displayed by various healers is similar in terms of the number of medicinal specimens (23 plants on average), with the exception of Hajia Bintu Camará, who only mentioned eight plants. The treatments performed by Hajia Bintu, an elderly woman who had been a midwife, were essentially intended for the village members, especially pregnant women and children. Her medicinal practice relied on her skills and reputation as a midwife, having perfected herself in the prophylactic use of medicinal plants on pregnant women and new-born infants^2^.

In his turn, Usumane Cassamá was still a young man when we first started our investigation, still in the first stages of his practice as a healer. He had learnt the use of plants from his father, who had meanwhile passed away, and started being called upon to prepare medicines, mostly by his village relatives. In the course of the following years, his reputation grew. All the other healers - Arafan Sané, Dgibi Camará, Fatu Bangorá and Tcherno Camará -, were already celebrated healers who treated patients from different villages or even distant regions.

Learning the ability to heal using plants develops differently in each case. Some healers start by learning those medicinal uses of plants known as “household remedies”, since they constitute the secret of a given lineage. The efficacy of these medicines is imbued with an ancestral symbolic significance, as will be shown below.

But there is also another way to obtain knowledge of medicinal plants: learning from other healers. Any individual, of any age or gender can seek a healer and ask to be apprenticed, which he/she may agree to in return for a fee, whether in money or goods. Healers who know certain household remedies can also increase their knowledge by learning new skills, thus increasing the number of diseases they can cure.

Thus, specialization derives from an individual’s expertise in the treatment of specific diseases with the use of certain plants, in addition to possessing specific knowledge on the combination of vegetable ingredients to make medicines. As healer Arafan Sané would say: “All plants are medicinal. All plants are good for curing, we need to know them, some people know some plants, and other people know others”.

Here are three examples of medicinal plants that, like many others, were used in various ways by different healers:

- *n’fankok* (*Paullinia pinnata*) used to treat “child weakness” (Fatu Bangorá), to fight scabies (Usumane Cassamé), and in the treatment of child diarrhea (Hajia Bintu Camará).
- *n’tonkindjá* (*Ficus sur*) used for lower back aches (Arafan Sané), failure to menstruate (Fatu Bangorá) and for stomach aches (Tcherno Camará e Dgibi Camará).
- *n’fol* (*Sarcocephalus latifolius*) used against intestinal parasites (Arafan Sané), constipation (Hajia Bintu Camará), stomach aches (Tcherno Camará e Dgibi Camará) and in the treatments that accompany pregnancy (Fatu Bangorá).

In terms of plant-picking habits, all healers follow a common principle: harvesting the vegetable elements needed for preparing medicine causing the least possible damage to the plants. Thus, while picking roots for instance, only a sufficient portion of the root is cut so as not to compromise the plant’s survival, just as in the collection of tree bark, the least damaged barks are chosen. These practices are just as much in accordance with a practical concern with the preservation of natural resources, as with an ontological respect for non-human beings, providing a basis for sustainability and contributing to the preservation of biodiversity.

The collection of the medicinal plants used by Tcherno Camará and Dgibi Camará was carried out together with both healers. However, as it will soon become clear, the symbolical proceedings involved in the treatments used by each of these two healers were totally different, since Tcherno Camará relied on pre-Islamic symbolical elements while Dgibi Camará is a *marabout* and thus followed Muslim practices.

We can therefore claim to have found two kinds of specialization; one specialization consisting in the knowledge of the medicinal characteristics of plants, and the another in the symbolic efficiency of the treatments that implied the use of plants.

### Protection mothers and children – healer Fatu Bangorá

The healer Fatu Bangorá is an old woman who specialized in assisting pregnant women and curing children’s diseases, besides treating a large number of adult ailments. She learned some of these medicines from her mother, but there was a specific episode that drew her to this activity. Her mother became gravely ill, and a healer from different ethnic group (Sussu), who lived the Republic of Guinea and was visiting the region, was the only one to hit upon the right treatment. When she saw this, Fatu decided to “buy his medicines”, or in other words to learn from him in exchange for payment in goods. From that moment on, she has been collecting different knowledge on medicinal plants from many other healers, not the least because, as she explained, she “had many children and sickness can fall upon us unexpectedly”.

She uses a broad range of plants (Table 1). One example concerns the prevention of the illness called *foie kumbé* (“big wind”). In this situation, women “expel blood during pregnancy and later die during childbirth, along with the infant” (very likely corresponding to situations in which the placenta is not securely attached). She uses the leaves of *mékinha* (*Crossopteryx febrifuga*), *n’sansan* (*Cassia sieberiana*), *n’sokor* (*Vitex madiensis*) and *urém* (*Landolphia dulcis*), dried and macerated in cold water. The resulting liquid is drunk by the women daily, starting in the third month of pregnancy.

When the baby is born, it is washed with the same medicine and also begins to drink it. When the breastfeeding period ends, the mother takes the child to the house of the healer, who will proceed with a symbolic ceremony. In this so-called “law of the remedy”, Fatu cuts off a small lock of the child’s hair, thus marking the end of the preventive period. As an expert in the treatment of childhood diseases, the pregnant mothers assisted by her, essentially make use of her services when their children become ill.

Despite not belonging to a lineage of traditional medicine experts, this healer gained recognition. The means by which she acquired knowledge of medicinal plants was not a ritual learning, neither was it based on any kind of magical initiation. Nevertheless, there are symbolical elements connected with some of the treatments, and they are marked by ritualistic practices, as in the example shown.

### The symbolic efficacy of *mesinhos di casa* – healers Arafan Sané and Tcherno Camará

Some healers anticipate and cure with *mesinhos di casa* (“household remedies”) – secrets about the use of certain plants that are passed down from parents to children, from grandparents to grandchildren. Arafan Sané and Tcherno Camará, now deceased, were two such healers. As such, they shared their knowledge of medicinal plants, but only within the boundaries of what they considered not to have a secret character. In spite of this, after several months of fieldwork, they explained the ritual procedures that accompanied most of his treatments. And they also revealed their fear that, in light of current cultural changes, their knowledge would not endure in future generations.

**Fig. 2:**
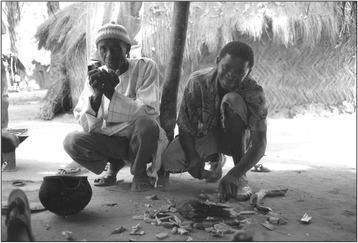
The healer Arafan Sané and his son/apprentice (Iafai Sané) preparing medicine with the root of *Bridelia micrantha*

Arafan Sané earned recognition for his ability to cure intestinal problems, as well as female reproductive health, and his reputation was based on the fact that he could cure female infertility. One of the main plants he used was the *n’tak* (*Bridelia micrantha*). Its root was collected, scraped and cut into pieces, which were placed in a pot (Fig. 2). The first pieces placed in this manner should add up to seven and were accompanied by a prayer. The preparation of the medicine consisted in boiling; it was taken by the ill every day in a small gourd. Treatment could last for a month or more, depending on the progress of the patient’s condition.

Such medicines must be prepared at home to be efficient, because their strength and efficacy lies in their having been supplied to humans by supernatural beings. Phytotherapeutic knowledge belongs to a given lineage, who obtained it in exchange with the supernatural beings (*iráns*).

The first inhabitants of a region, the founders or so-called “land owners”, gained political dominance by having been the first to inhabit a determined area. However, this political dominance is ideologically legitimized by a process of symbolic appropriation of the space, resulting from the imaginary idea that they had accomplished an initial exchange with the supernatural beings [31, 32].

The accounts of initial exchange coincide entirely – according to the oldest men:

“The founders took the land from the irán’s hands, offering a male nephew who has still not known any woman, and a female niece already full breasted, but who has not yet known any man”.

In exchange, they received protection of the supernatural beings – protection for men and for the natural space. They were also given knowledge of plants:

“Household remedies were handed down by the iráns. Our ancestors asked them for plants to cure themselves and their families. These plants are dangerous because ancestors payed dearly for them; in that time they bought much beverage and killed much game. If you start picking these plants without paying for them, you are starting a war with the iráns.” (Arafan Sané)

**Fig. 3:**
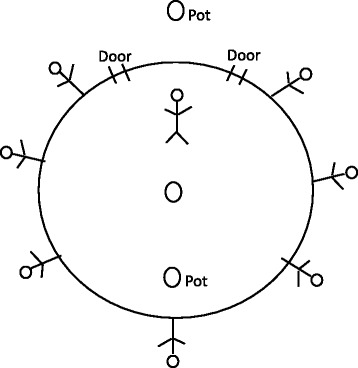
Drawing by the healer Arafan Sané, describing the disposition of supernatural beings in local territory

According to this account, the *iráns* would have lived in the territory founded by the lineage ancestors; they were entities in whose name the healer worked to make his treatments produce the desired effects. This healer was supported by seven *iráns*. To explain this he used a drawing (Fig. 3) where they are shown standing around the “pots laid out for them to drink”.

The figures he started drawing represent the *iráns* who protect the territory. The most important *irán*, Baken Kantchadáber, was the one with whom Arafan’s ancestors did the mythical initial trade, and who lives near the neighboring village of Caontchinque, in a tree known as *n’kinan* (unidentified). It is he who presided to the nalu’s masculine initiation rite (before Islamization), and continues to embody a talisman (cotton yarn) protecting people from the evils that may arise within Nalu territory. Around Baken Kantchadáber stood the “Guardian *iráns*”: Mangbenkar, his son, in the Canábene forest by the river, living in a small tree *n’koia,* (Cf. *Synsepalum pobeguinianum*) where a large snake or swarm of bees are often seen; Banam, near the village of Arafan (Sogobol), in a huge *n’kauuê* (kapok; *Ceiba pentandra*); N’taxanból, in an orchard near the village of Sogobol, in a *n’tisebá* tree (*Pterocarpus santalinoides*); Iaulin, near the village of Catomboi, in a termite mound; Kondor, in the area of Iembérem, in a large *n’kauuê*; and Tukudu, also in Iembérem, in a large *n’bim* (*Dialium guineense*)^3^. Just as in human society, Mangbenkar would have occupied his father’s place, given the latter’s loss of interest in the position due to old age. The two doors represent the openings through which the *iráns* make their entrance. In this drawing, the figure represented inside the circle would be Mangbenkar. When the healer was on a journey to treat sick people in other villages, he would summon the resident *iráns* in these areas. For example, when he went to Iembérem, he called for Kondor and Tukudu’s help. In his words, he “worked with both of them”.

The power of “household remedies” therefore resulted from the efficiency invested on them by supernatural entities, in a process through which the healers possess the knowledge of their ancestors, but derive their power from the genies^4^.

In his turn, Tcherno Camará was a nurse in a Health center in the region, and at the same time was a healer, an agent of traditional medicine. Thus, he was simultaneously an agent of two distinct medical systems. He learned to cure with plants from his grandfather, an expert *djambakus*; and trained to be a nurse in a public nursing course taken after Guinea-Bissau’s independence (1974). The knowledge of modern medicine that he brought to his healer activity was mainly in terms of Portuguese disease terminology. Patients appeared at his doorstep, mostly seeking a cure for malaria, venereal diseases, “belly” aches or eye diseases. Men also commonly brought up impotence problems, while women sought a cure for lack of menstruation and infertility. To solve all these conditions, he resorted to a wide range of plants (some of which are described in Table 1).

Arafan and Tcherno worked in the name of the supernatural beings, so that the plants would achieve the desired ends, so that the *iráns* should grant him efficacy. Their skills as healers derived from the symbolic power consolidated from their ancestors.

Intervention by the *iráns* is associated with the presence of ritual objects that have become known as the sculptures of the Nalu people [31, 33]. One of these, designated as *Numba* (or *Nimba*), is a sculpture which may assume two different shapes: one resembles the bust of a woman, and the other – which is predominant in the region in question - resembling a turtle, whose function is to protect houses and their inhabitants, with their power also bearing influence on the fertility of women and fields. The other, called *N’tomon* (also called *Matchol* in other regions, the name by which this sculpture is known in African Art markets), has the appearance of a stylized bird, and is connected with pre-Islamic rituals used to solve matters of justice, requests for individual blessings (such as wealth or descent) and also governs matters connected with the fate of lineages.

These healers were also ritual masters, and therefore the efficiency of their treatments was not based solely on their knowledge of the features of vegetable medicines, but also on the strength that *N’tomon* conferred upon them. Many of these sculptures disappeared with colonization, some of them being initially appropriated by European museums or African Art markets, and later losing their importance with Islamization. Unlike Tcherno, who owned several *N’tomon* sculptures, in Arafan’s village there no longer existed any sculptures. Nevertheless, he maintained the rituals of pre-Islamic origin that conferred symbolical efficiency to his treatments, even carrying them out on the site where a sculpture had previously stood. Today, both of their children continue their work. Arafan’s son, Iafai Sané, uses his knowledge of medicinal plants learned from his father in the treatment of patients from neighboring villages. Given that he was still young when his father died - thus not having completed his initiation in Nalu ritual proceedings, and consequently not having mastered all the secrets -, he is only fit to perform part of the ritual tasks. Tcherno’s son, Amará Camará, substitutes his father completely, becoming locally acknowledged healer and ritual master.

The religious transformations inherent to the growing importance of Islam, and the cultural changes implied by the modernization of Guinea-Bissau’s economic standards, have marked the life of the Nalu over the last decades [24, 34, 35], leading to the disappearance of ritual objects and the beliefs attached to them. However, the continuance of these healers’ work and knowledge through their sons shows that cultural shifts are not operated abruptly, but in a process of resilience rather than rupture.

### The power of sacred writing on plants - *marabout* Dgibi Camará

As a result of Islamization, the use and the symbolic perception of plants has become distinct from pre-Islamic perception. However, these two different worldviews coexist, and the infirm often follow parallel therapeutic courses, resorting simultaneously to pre-Islamic medicine healers and *marabouts*, as well as to the local nurses working for the healthcare system. In other words, “the whole symbolical and therapeutic range is used to ensure effectiveness of the cure. The different axioms are not perceived as contradictory, and the only governing rationale is that of the cure” ([16], p. 115). On the other hand, the syncretic nature of African Islam (e.g. [36, 37]) leads to a crossing between supernatural entities and conceptions of natural elements.

*Marabouts* perform different activities. They are educated in the complex Islamic mystical system; in numerology and astronomy (e.g. [38–41]). They act as healers, combining a therapeutic based on medicinal plants, with divination and Islamic prayer. Their therapeutic activity is thus combined with magical activity, although just like other local medicine agents they become specialized in the treatment of certain diseases, particularly those with a magic-religious etiology. But the activity of *marabouts* can also contain an element of negative magic, since they are trained to cast curses, including diseases.

The medical practices of these *marabouts* differ from those of other healers, not just because the treatments are always accompanied by Quranic prayers, but also because the medicines used in many of them are prepared from vegetable elements mixed with *nás* (sacred water obtained by washing a wooden tablet on which passages of the Quran are inscribed with vegetable pigments)^5^.

Dgibi Camará underwent a long Quranic education, and his use of medicinal plants concurs with the Muslim practice system. He was the pupil of a Muslim master from a different ethnicity (Djacanca), from whom he learned the Islamic writing, religion and magic, as well as the uses of many medicinal plants. Nevertheless, his knowledge was completed with extensive teachings in traditional medicine from an old Nalu healer.

This *marabout* has some simple uses for medicinal plants that don’t imply ritual procedures. *N’sansan* (*Cassia sieberiana*), for instance, is used for body aches. However, most medicines are prepared with *nás.* As he explained:

“A medicine is mixed with God’s name, with the Quran’s name; the *nás* is made, given to the sick person, who then makes an infusion with the medicine: it is boiled and the *nás* is added, and then it is drunk. It is like when you cook rice on which you put the garnish. The medicine is the rice, and the *nás* is the garnish. If you cook only the rice, you can eat it but it has no flavor, but when you add the *nás*, the medicine increases its power.”

Thus, the *nás* increases the power of *n’bukui* bark (*Bauhinia thonningii*) used, amongst other things, to cure hemorrhoids; or *n’sópsó*p leaves (*Annona muricata*) used for stomach aches, or even the treatment of swollen limbs (probably Filariasis) using parts of several plants such as *n’txamborta* (*Diospyros heudelotii*) and *n’fakat* (*Hibiscus sterculiifolius*).

As a *marabout*, Dgibi is also a specialist in the treatment of diseases with religious or magical causes. In Islamic terms, these diseases are attributed to the intervention of genies (*jinn*) and *setani* (*satan*), the name for devil, as witnessed among other African Muslim societies (e.g. [42, 43]). The descriptions, both of the symptoms and of the facts that caused them, are all but identical to the reports of the diseases caused by the *iráns* in the nosology of pre-Islamic origin. These diseases result from the sexual desire of *setani* or from breaching a contract made with one of these supernatural beings. There are also suitable medicines and *nás* for these diseases.

Therefore, the perception of diseases is similar amongst healers and *marabouts*, as well as amongst the sick who seek their services. Nevertheless, with the progressive Islamization there has been, on the one hand, a reinterpretation of non-natural etiologic categories and the substitution of pre-Islamic supernatural entities by Islamic genies [16, 44]. On the other hand, we have seen an increasing demand for *marabouts* to the detriment of masters who follow traditional pre-Islamic rituals, especially in the treatment of diseases of magical-religious nature.

## Conclusion

This study demonstrated the importance of symbolic aspects in the use of medicinal plants, agreeing with the conclusions reached by other studies on the treatment of sickness in West Africa, as for example the work by Fainzang, where it is stated that “(…) the therapeutic discourse affirmed the treatment’s validity, leading to eventual intervention by the sacred to legitimate it; the cure was more recommended for its magic (symbolic) value than for its intrinsic value” ([45], p. 420).

In the pre-Islamic worldview prevailing within Nalu’s traditional phytotherapy system, the concept of medicinal plants is part of a specific cosmology. In general, natural beings, whether animals or plants, are seen as elements of the world at the same level as humans in a holistic and systemic vision of the universe [46]. The different cosmologic elements are understood not as neutral forces, but as vital essences. As in other African contexts the agency of supernatural entities is similarly acknowledged (e.g. [47, 48]). Humans, plants, and supernatural entities (*iráns*) participate in a complex network of social relations.

It can be stated that amongst the Nalu, medicinal plants occupy a different place in the pre-Islamic medicine and in the medicine practiced by Islamic masters. Knowledge of plants’ pharmacological action will be similar in every respect, but they differ in the symbolical means used to render them efficient. The Muslim practitioner seizes divine energy, allowing himself to be penetrated by its supernatural efficiency. It is easy to understand that this quasi-sacramental value conceded to the reading of the Quran has drifted to more practical uses since the first generations of Islam, and that the versicles have come to be used for purposes of healing or divination [40, 49]. The presence of sacred words in medicine is “a direct way of infusing the body with the sacred liturgy, yet another kind of embodiment of the text and a textualization, hence sanctification, of the body” ([50], p. 27).

In the healing practices based on pre-Islamic conceptions, plants are mediators for the power of *iráns*. Given that plants with beneficial properties were an offering from the *iráns* to the forefathers, plants themselves possess a magical and symbolical power. Plants’ medicinal value stems from a bond established through human lineages, which includes both the living and their ancestors [51], in a line of continuity with the supernatural sphere.

Within Muslim practices, medicinal plants become simply a material instrument, and the magical effect lies with the reproduction of the sacred text, in a direct divine intervention. Although the *marabout*’s power is also mediated by the strength of Islamic genies, these do not possess the autonomy of pre-Islamic genies. Thus, it is as if the material means found in the vegetable elements had lost part of their symbolic meaning and value to the power of the new medium afforded by the sacred writings^6^.

In conclusion, in the Nalu healing systems under analysis, we found a similar degree of knowledge and use of medicinal plants amongst healers who follow a non-Islamic cosmology and healers who perform Islamic ritualistic practices. Distinctions in the use of medicinal plants are drawn according to the healers’ field of expertise, gender, personal history and individual learning careers. Nevertheless, the non-Muslim and Muslim procedures involved in the bestowal of symbolic efficacy on treatments differ and are supported by distinct ontologies: to the former, plants possess an intrinsic value derived from the bonds linking human and superhuman entities, while for the latter plants relinquish that value to the relation established directly by humans with God, mediated by Holy Scriptures.

## Footnotes

1 Other studies report the traditional medical systems (notions of disease, treatments and diagnostics) without any reference to medicinal plants, of Manjack [47, 52] and Felupe/Djola people [53].

2 In fact, Hajia Bintu Camará’s specialty is locally known as midwifery, and not exactly as a healer. Her medicinal practices and the social recognition that her knowledge received were only revealed to me through daily observations, which explains their inclusion in this study.

3 The sites where *iráns* live are considered sacred and as such are preserved. These sacred forests have recently been considered protected areas, in a process that can be described as a reinterpretation of their meaning or as a commodification of nature [46].

4 Although the power of healers derives from the genies, like in other African cultures e.g. [54], there is no possession ritual.

5 The use of dissolved written Quran verses in water as preventive or curative treatments is common in many Islamic contexts (e.g. [41, 50, 55]).

6 In other African contexts marked by the expansion of the African Independent Churches, we can witness a similar process. Even though the agents of these churches may possess a thorough knowledge of medicinal plants equal to traditional healers [56], their understanding of the curing processes are not concordant, giving rise to a conflict between the treatments performed by traditional healers and the treatments performed in these churches by their prophets or pastors. For instance, in South Africa and Mozambique, the Zionist [57, 58], and Pentecostal churches [59, 60] reject the influence of the spirits from traditional African religions on the curing process, the spiritual dimension of the treatment being transferred to the Holy Spirit, through biblical texts and prayers.
